# *catena*-Poly[[di­aqua­[μ_2_-4-(4-carb­oxy­phen­oxy)benzoato](μ_2_-4,4′-oxydibenzo­ato)praseodymium(III)] monohydrate]

**DOI:** 10.1107/S1600536813026421

**Published:** 2013-10-05

**Authors:** Ping Li, Duo-Meng Su, Chang-Ge Zheng

**Affiliations:** a School of Chemical and Material Engineering, Jiangnan University, 1800 Lihu Road, Wuxi, Jiangsu Province 214122, People’s Republic of China

## Abstract

In the title compound, {[Pr(C_14_H_8_O_5_)(C_14_H_9_O_5_)(H_2_O)_2_]·H_2_O}_*n*_, the Pr^III^ cation is eight-coordinated by six carboxyl O atoms from both a monoanionic 4-(4-carb­oxy­phen­oxy)benzoate and a dianionic 4,4′-oxydibenzoate ligand (four bridging with two from a bidentate chelate inter­action), and two O-atom donors from water mol­ecules. A single water mol­ecule of solvation is also present. The complex units are linked through carboxyl *O*:*O*′ bridges giving a two-dimensional sheet polymer lying parallel to (001). An overall three-dimensional network structure is generated through inter­molecular carb­oxy­lic acid and water O—H⋯O hydrogen bonds and weak C—H⋯O inter­actions.

## Related literature

For the potential properties of metal-organic complexes involving polycarboxyl­ate ligands, see: Li *et al.* (2011 ▶); Wang *et al.* (2004 ▶, 2005 ▶); Lin *et al.* (2010 ▶); Sun *et al.* (2009 ▶); Xu *et al.* (2011 ▶); Łyszczek & Mazur (2012 ▶). For similar structures, see: Thirumurugan & Natarajan (2004 ▶); Zhang *et al.* (2005 ▶).
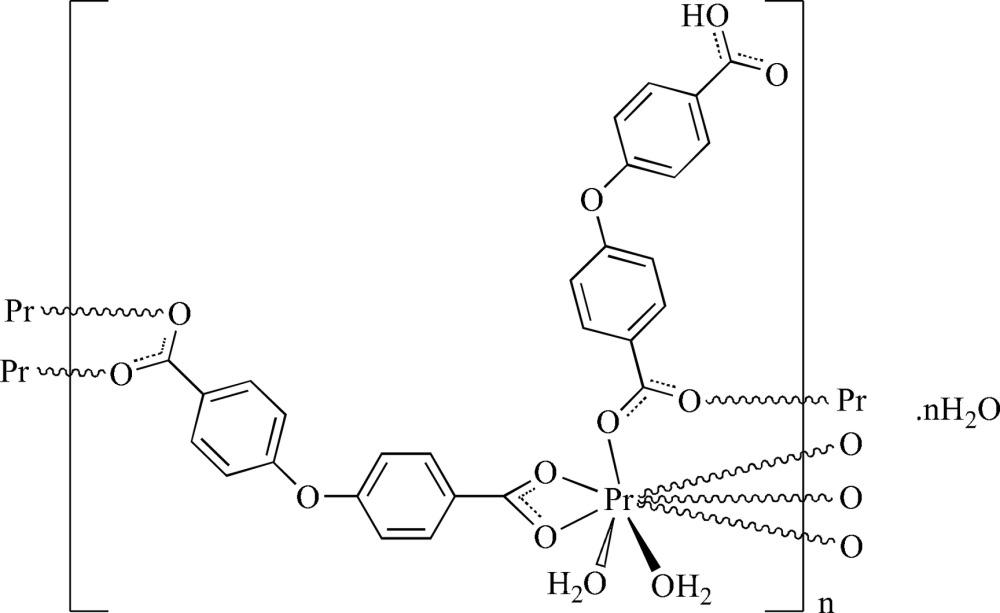


## Experimental

### 

#### Crystal data

[Pr(C_14_H_8_O_5_)(C_14_H_9_O_5_)(H_2_O)_2_]·H_2_O
*M* *_r_* = 708.37
Monoclinic, 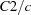

*a* = 27.3970 (17) Å
*b* = 9.5764 (6) Å
*c* = 21.6754 (14) Å
β = 97.433 (1)°
*V* = 5639.1 (6) Å^3^
*Z* = 8
Mo *K*α radiation
μ = 1.80 mm^−1^
*T* = 296 K
0.21 × 0.16 × 0.15 mm

#### Data collection

Bruker SMART CCD area-detector diffractometer
Absorption correction: multi-scan (*SADABS*; Bruker, 1997 ▶) *T*_min_ = 0.704, *T*_max_ = 0.774
20449 measured reflections
4972 independent reflections
4498 reflections with *I* > 2σ(*I*)
*R*_int_ = 0.026

#### Refinement

*R*[*F* ^2^ > 2σ(*F* ^2^)] = 0.025
*wR*(*F* ^2^) = 0.059
*S* = 1.08
4972 reflections
381 parameters
H-atom parameters constrained
Δρ_max_ = 0.59 e Å^−3^
Δρ_min_ = −0.51 e Å^−3^

### 

Data collection: *SMART* (Bruker, 1997 ▶); cell refinement: *SAINT* (Bruker, 1997 ▶); data reduction: *SAINT*; program(s) used to solve structure: *SHELXS97* (Sheldrick, 2008 ▶); program(s) used to refine structure: *SHELXL97* (Sheldrick, 2008 ▶); molecular graphics: *SHELXTL* (Sheldrick, 2008 ▶); software used to prepare material for publication: *SHELXTL*.

## Supplementary Material

Crystal structure: contains datablock(s) I, New_Global_Publ_Block. DOI: 10.1107/S1600536813026421/zs2278sup1.cif

Structure factors: contains datablock(s) I. DOI: 10.1107/S1600536813026421/zs2278Isup2.hkl

Additional supplementary materials:  crystallographic information; 3D view; checkCIF report

## Figures and Tables

**Table 1:** Selected bond lengths (Å)

Pr1—O9^i^	2.3983 (18)
Pr1—O5	2.4105 (19)
Pr1—O12^ii^	2.412 (2)
Pr1—O8^iii^	2.4692 (19)
Pr1—O11	2.4719 (18)
Pr1—O10	2.5152 (19)
Pr1—O1	2.5163 (19)
Pr1—O7^iii^	2.6534 (19)

Symmetry codes: (i) 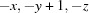; (ii) 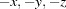; (iii) 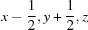.

**Table 2:** Hydrogen-bond geometry (Å, °)

*D*—H⋯*A*	*D*—H	H⋯*A*	*D*⋯*A*	*D*—H⋯*A*
O3—H3⋯O1^iv^	0.82	2.02	2.822 (3)	166
O10—H10*B*⋯O2^v^	0.85	2.09	2.880 (3)	154
O11—H11*A*⋯O4^vi^	0.84	1.84	2.683 (3)	176
O11—H11*B*⋯O8^vii^	0.84	1.89	2.707 (3)	163
C9—H9⋯O3^vi^	0.93	2.48	3.337 (4)	153
C25—H25⋯O13^iii^	0.93	2.59	3.454 (7)	155

Symmetry codes: (iii) 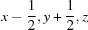; (iv) 
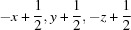
; (v) 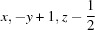; (vi) 
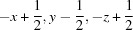
; (vii) 
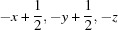
.

**Fig. 1.**
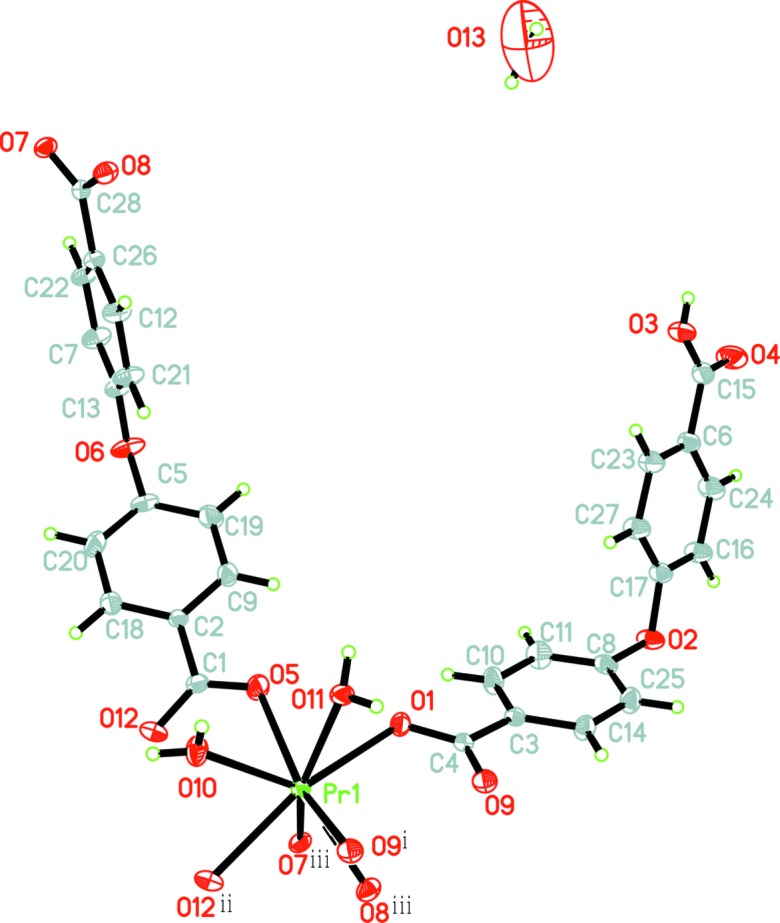
The asymmetric unit of the title complex showing 30% probability displacement ellipsoids and the atom-numbering scheme. For symmetry codes: (i) -x, -y +1, -z; (ii) -x, -y, -z; (iii) x -1/2, y +1/2, z.

**Fig. 2.**
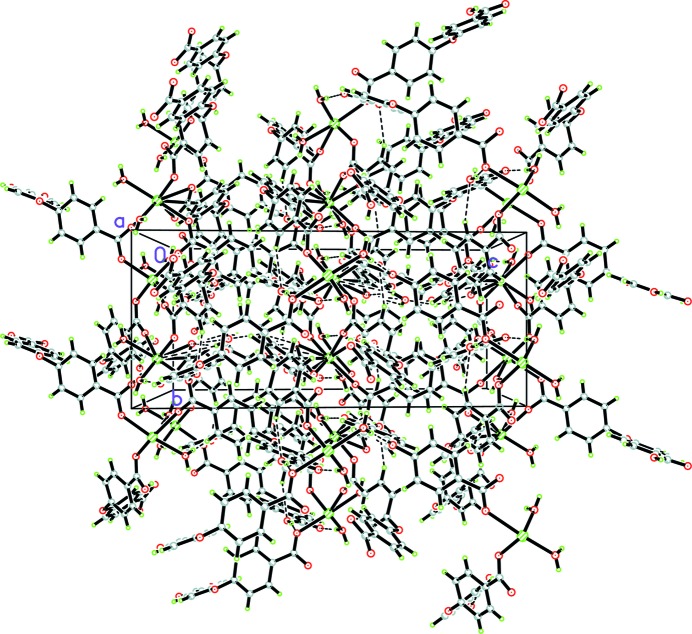
A packing diagram for the three-dimensional structure formed through O—H···O and C—H···O hydrogen-bonding interactions. Hydrogen bonds are indicated by dashed lines.

## supplementary crystallographic information

### 1. Comment

Metal–organic frameworks (MOFs) with lanthanides have attracted much attention because of their abundant structural chemistry and valuable optical and magnetic properties (Lin *et al.*, 2010). In contrast to coordination polymers with other transition metals, the architecture of lanthanide coordination polymers is hard to control owing to large coordination numbers and flexible coordination geometries of the lanthanide atom. The ligand 4-(4-carboxyphenoxy)benzoic acid (H_2_oba) can be deprotonated, giving Hoba^-^ or oba^2-^ species and as *V*-shaped ligands, they can offer more many coordination modes compared to a linear ligand. Owing to the nonlinear flexibility around the etheric oxygen, this ligand can readily generate helical coordination polymers (Łyszczek & Mazur, 2012).

In the title praseodymium(III) complex with 4-(4-carboxyphenoxy)benzoic acid, {[Pr(C_14_H_11_O_6_)(C_14_H_10_O_6_)(H_2_O)_2_] . H_2_O}_n_, the Pr^III^ cations have irregular eight-coordinate stereochemistry, the asymmetric unit comprising one Pr^III^ cation, an an Hoba^-^ ligand, an oba^2-^ ligand, two monodentate water molecules (O10 and O11) and one water molecule of solvation (O13) (Fig. 1). There are two types of coordination modes with the oba^2-^ and Hoba^-^ ligands: (*a*) two carboxylate groups of the oba^2-^ ligand adopt a bridging bidentate mode (O5, O12^ii^) and a bidentate chelate mode (O7^iii^, O8^iii^), respectively, connecting three Pr^III^ atoms; (*b*) one carboxylate group of the Hoba^-^ ligand adopts a bridging bidentate (O1, O9^i^) mode, connecting two Pr^III^ atoms. For symmetry codes, see Table 1. The carboxylic acid group (O4, O3) is un-coordinated. The Pr—O bond lengths [range 2.3983 (18)–2.6534 (19) Å] (Table 1) are comparable with those in similar Pr^III^ complexes (Thirumurugan & Natarajan, 2004; Zhang *et al.*, 2005). A two-dimensional coordination polymer is generated, lying parallel to (0 0 1). Adjacent layers are joined into a three-dimensional framework structure (Fig. 2) through intermolecular carboxylic acid and water O—H···O hydrogen bonds and weak C—H···O hydrogen-bonding interactions (Table 2).

### 2. Experimental

A mixture of 4-(4-carboxyphenoxy)benzoic acid (0.026 g, 0.1 mmol), Pr(NO_3_)_3_ 6H_2_O (0.15 mmol, 62.2 mg), and deionized water (8 ml) was sealed in a teflon-lined stainless steel vessel (25 ml) and heated at 433 K for 72 h. The vessel was then cooled slowly to room temperature. Green block-like crystals were obtained by filtration and washed with water. Yield: 50.4 mg (47.5%, based on Pr). Elemental analysis: calcd. for C_28_H_23_O_13_Pr, C 47.59%, H 3.25%. Found: C 47.48%, H 3.19%.

### 3. Refinement

C-Bound H atoms were were placed in calculated positions and were treated as riding, with C—H = 0.93 Å, with *U*_iso_(H) = 1.2*U*_eq_(C). O-Bound H-atoms were also placed in calculated positions (O—H = 0.82–0.85 Å) and were allowed to ride with *U*_iso_(H) = 1.2–1.5*U*_eq_(O).

### Crystal data

**Table:**

[Pr(C_14_H_8_O_5_)(C_14_H_9_O_5_)(H_2_O)_2_]·H_2_O	*F*(000) = 2832
*M**_r_* = 708.37	*D*_x_ = 1.669 Mg m^−^^3^
Monoclinic, *C*2/*c*	Mo *K*α radiation, λ = 0.71073 Å
Hall symbol: -C 2yc	Cell parameters from 9984 reflections
*a* = 27.3970 (17) Å	θ = 2.4–27.6°
*b* = 9.5764 (6) Å	µ = 1.80 mm^−^^1^
*c* = 21.6754 (14) Å	*T* = 296 K
β = 97.433 (1)°	Block, green
*V* = 5639.1 (6) Å^3^	0.21 × 0.16 × 0.15 mm
*Z* = 8	

### Data collection

**Table:**

Bruker SMART CCD area-detector diffractometer	4972 independent reflections
Radiation source: fine-focus sealed tube	4498 reflections with *I* > 2σ(*I*)
Graphite monochromator	*R*_int_ = 0.026
φ and ω scans	θ_max_ = 25.0°, θ_min_ = 1.9°
Absorption correction: multi-scan (*SADABS*; Bruker, 1997)	*h* = −32→27
*T*_min_ = 0.704, *T*_max_ = 0.774	*k* = −11→11
20449 measured reflections	*l* = −25→25

### Refinement

**Table:**

Refinement on *F*^2^	Primary atom site location: structure-invariant direct methods
Least-squares matrix: full	Secondary atom site location: difference Fourier map
*R*[*F*^2^ > 2σ(*F*^2^)] = 0.025	Hydrogen site location: inferred from neighbouring sites
*wR*(*F*^2^) = 0.059	H-atom parameters constrained
*S* = 1.08	*w* = 1/[σ^2^(*F*_o_^2^) + (0.0163*P*)^2^ + 15.8309*P*] where *P* = (*F*_o_^2^ + 2*F*_c_^2^)/3
4972 reflections	(Δ/σ)_max_ = 0.001
381 parameters	Δρ_max_ = 0.59 e Å^−^^3^
0 restraints	Δρ_min_ = −0.51 e Å^−^^3^

### Special details

**Table:**

Geometry. All e.s.d.'s (except the e.s.d. in the dihedral angle between two l.s. planes) are estimated using the full covariance matrix. The cell e.s.d.'s are taken into account individually in the estimation of e.s.d.'s in distances, angles and torsion angles; correlations between e.s.d.'s in cell parameters are only used when they are defined by crystal symmetry. An approximate (isotropic) treatment of cell e.s.d.'s is used for estimating e.s.d.'s involving l.s. planes.
Refinement. Refinement of *F*^2^ against ALL reflections. The weighted *R*-factor *wR* and goodness of fit *S* are based on *F*^2^, conventional *R*-factors *R* are based on *F*, with *F* set to zero for negative *F*^2^. The threshold expression of *F*^2^ > σ(*F*^2^) is used only for calculating *R*-factors(gt) *etc*. and is not relevant to the choice of reflections for refinement. *R*-factors based on *F*^2^ are statistically about twice as large as those based on *F*, and *R*- factors based on ALL data will be even larger.

### Fractional atomic coordinates and isotropic or equivalent isotropic displacement parameters (Å^2^)

**Table:**

	*x*	*y*	*z*	*U*_iso_*/*U*_eq_	
Pr1	0.014443 (5)	0.250000 (14)	0.008512 (7)	0.02218 (6)	
C1	0.08267 (9)	−0.0154 (3)	0.06113 (13)	0.0281 (6)	
C2	0.12798 (9)	−0.0851 (3)	0.09282 (12)	0.0257 (6)	
C3	0.05948 (9)	0.5746 (3)	0.17173 (12)	0.0258 (6)	
C4	0.04362 (9)	0.5108 (3)	0.10933 (12)	0.0254 (6)	
C5	0.21188 (10)	−0.2111 (3)	0.15102 (14)	0.0341 (7)	
C6	0.26088 (11)	0.8308 (3)	0.39077 (15)	0.0377 (7)	
C7	0.33610 (11)	−0.3437 (3)	0.18593 (14)	0.0397 (7)	
H7	0.3323	−0.3976	0.2207	0.048*	
C8	0.09428 (10)	0.6972 (3)	0.28360 (13)	0.0327 (6)	
C9	0.15967 (11)	−0.0134 (3)	0.13644 (16)	0.0418 (8)	
H9	0.1527	0.0785	0.1459	0.050*	
C10	0.08283 (12)	0.4950 (3)	0.22016 (14)	0.0401 (7)	
H10	0.0867	0.3995	0.2148	0.048*	
C11	0.10043 (13)	0.5567 (4)	0.27647 (14)	0.0447 (8)	
H11	0.1162	0.5033	0.3089	0.054*	
C12	0.34745 (11)	−0.1805 (3)	0.08407 (14)	0.0378 (7)	
H12	0.3513	−0.1246	0.0500	0.045*	
C13	0.29695 (11)	−0.2675 (3)	0.15686 (14)	0.0324 (7)	
C14	0.05244 (12)	0.7155 (3)	0.18124 (14)	0.0365 (7)	
H14	0.0355	0.7690	0.1496	0.044*	
C15	0.31376 (12)	0.8545 (3)	0.41214 (17)	0.0430 (8)	
C16	0.17743 (11)	0.8314 (4)	0.41406 (14)	0.0432 (8)	
H16	0.1548	0.8484	0.4417	0.052*	
C17	0.16187 (11)	0.7811 (3)	0.35461 (14)	0.0333 (7)	
C18	0.13862 (12)	−0.2214 (3)	0.07976 (17)	0.0437 (8)	
H18	0.1172	−0.2720	0.0513	0.052*	
C19	0.20161 (12)	−0.0762 (4)	0.16618 (16)	0.0459 (8)	
H19	0.2225	−0.0279	0.1960	0.055*	
C20	0.18108 (13)	−0.2837 (4)	0.10883 (18)	0.0492 (9)	
H20	0.1884	−0.3754	0.0994	0.059*	
C21	0.30232 (11)	−0.1854 (4)	0.10597 (15)	0.0413 (8)	
H21	0.2759	−0.1340	0.0866	0.050*	
C22	0.38089 (11)	−0.3398 (3)	0.16320 (14)	0.0357 (7)	
H22	0.4071	−0.3926	0.1823	0.043*	
C23	0.24464 (12)	0.7818 (4)	0.33152 (15)	0.0414 (8)	
H23	0.2672	0.7660	0.3037	0.050*	
C24	0.22675 (12)	0.8557 (4)	0.43135 (15)	0.0452 (8)	
H24	0.2374	0.8894	0.4710	0.054*	
C25	0.07024 (12)	0.7778 (3)	0.23706 (14)	0.0396 (7)	
H25	0.0660	0.8730	0.2430	0.048*	
C26	0.38714 (10)	−0.2577 (3)	0.11215 (14)	0.0284 (6)	
C27	0.19530 (12)	0.7562 (3)	0.31339 (15)	0.0404 (8)	
H27	0.1847	0.7224	0.2737	0.048*	
C28	0.43485 (10)	−0.2515 (3)	0.08701 (13)	0.0273 (6)	
O1	0.05551 (7)	0.3854 (2)	0.10037 (9)	0.0348 (5)	
O2	0.11178 (8)	0.7623 (2)	0.34028 (10)	0.0405 (6)	
O3	0.34277 (8)	0.8348 (3)	0.36883 (12)	0.0494 (6)	
H3	0.3715	0.8454	0.3840	0.074*	
O4	0.32852 (9)	0.8911 (3)	0.46497 (12)	0.0614 (7)	
O5	0.08076 (8)	0.1151 (2)	0.06299 (11)	0.0446 (6)	
O6	0.25350 (8)	−0.2782 (3)	0.18227 (11)	0.0452 (6)	
O7	0.46781 (7)	−0.3427 (2)	0.10035 (9)	0.0312 (4)	
O8	0.44170 (7)	−0.1528 (2)	0.04974 (10)	0.0350 (5)	
O9	0.01975 (7)	0.5838 (2)	0.06788 (9)	0.0328 (4)	
O10	0.04446 (8)	0.1241 (2)	−0.08121 (10)	0.0385 (5)	
H10A	0.0200	0.0888	−0.1040	0.058*	
H10B	0.0591	0.1815	−0.1024	0.058*	
O11	0.08179 (7)	0.38020 (19)	−0.03119 (9)	0.0326 (4)	
H11B	0.0752	0.4617	−0.0446	0.039*	
H11A	0.1106	0.3833	−0.0120	0.039*	
O12	0.04866 (7)	−0.0880 (2)	0.03345 (10)	0.0393 (5)	
O13	0.5392 (3)	0.6238 (7)	0.2092 (3)	0.251 (4)	
H13B	0.5098	0.5933	0.2009	0.377*	
H13A	0.5419	0.6668	0.1755	0.377*	

### Atomic displacement parameters (Å^2^)

**Table:**

	*U*^11^	*U*^22^	*U*^33^	*U*^12^	*U*^13^	*U*^23^
Pr1	0.01793 (9)	0.02186 (9)	0.02589 (10)	0.00108 (5)	−0.00048 (6)	0.00062 (5)
C1	0.0228 (14)	0.0318 (16)	0.0294 (15)	0.0028 (12)	0.0028 (11)	0.0000 (12)
C2	0.0199 (13)	0.0250 (14)	0.0317 (15)	0.0013 (11)	0.0016 (11)	0.0019 (11)
C3	0.0234 (14)	0.0281 (14)	0.0245 (14)	−0.0003 (11)	−0.0014 (11)	−0.0007 (11)
C4	0.0202 (13)	0.0269 (14)	0.0289 (14)	−0.0007 (11)	0.0020 (11)	−0.0017 (11)
C5	0.0213 (15)	0.0440 (17)	0.0377 (17)	0.0088 (13)	0.0071 (12)	0.0143 (14)
C6	0.0304 (16)	0.0357 (17)	0.0446 (18)	−0.0017 (13)	−0.0044 (13)	0.0023 (14)
C7	0.0328 (16)	0.0498 (19)	0.0375 (17)	0.0124 (14)	0.0086 (13)	0.0182 (15)
C8	0.0258 (15)	0.0468 (18)	0.0249 (15)	−0.0038 (13)	0.0009 (11)	−0.0083 (13)
C9	0.0391 (18)	0.0255 (15)	0.056 (2)	0.0058 (13)	−0.0112 (15)	−0.0054 (14)
C10	0.054 (2)	0.0275 (16)	0.0357 (17)	0.0031 (14)	−0.0073 (14)	0.0014 (13)
C11	0.056 (2)	0.0457 (19)	0.0278 (16)	0.0031 (16)	−0.0100 (14)	0.0035 (14)
C12	0.0316 (16)	0.0418 (18)	0.0407 (17)	0.0061 (13)	0.0074 (13)	0.0163 (14)
C13	0.0248 (15)	0.0407 (17)	0.0322 (16)	0.0070 (12)	0.0053 (12)	0.0079 (13)
C14	0.0419 (18)	0.0323 (16)	0.0322 (16)	0.0088 (13)	−0.0073 (13)	−0.0025 (13)
C15	0.0335 (17)	0.0340 (17)	0.058 (2)	0.0008 (14)	−0.0056 (16)	0.0016 (15)
C16	0.0330 (17)	0.064 (2)	0.0319 (16)	−0.0041 (15)	0.0032 (13)	−0.0085 (15)
C17	0.0280 (16)	0.0407 (17)	0.0298 (16)	−0.0009 (13)	−0.0012 (12)	−0.0018 (13)
C18	0.0370 (18)	0.0334 (17)	0.057 (2)	0.0043 (14)	−0.0082 (15)	−0.0103 (15)
C19	0.0370 (18)	0.0427 (19)	0.053 (2)	0.0028 (15)	−0.0151 (15)	−0.0004 (16)
C20	0.046 (2)	0.0336 (17)	0.065 (2)	0.0169 (15)	−0.0038 (17)	−0.0079 (16)
C21	0.0266 (16)	0.052 (2)	0.0455 (19)	0.0139 (14)	0.0052 (13)	0.0185 (16)
C22	0.0276 (15)	0.0398 (17)	0.0395 (17)	0.0119 (13)	0.0040 (13)	0.0103 (14)
C23	0.0320 (18)	0.052 (2)	0.0402 (18)	−0.0020 (14)	0.0054 (14)	−0.0041 (15)
C24	0.0392 (18)	0.058 (2)	0.0354 (17)	−0.0057 (16)	−0.0077 (14)	−0.0089 (15)
C25	0.048 (2)	0.0332 (16)	0.0358 (17)	0.0049 (14)	−0.0032 (14)	−0.0105 (13)
C26	0.0241 (15)	0.0284 (15)	0.0328 (16)	0.0021 (11)	0.0042 (12)	−0.0008 (11)
C27	0.0337 (18)	0.057 (2)	0.0291 (16)	−0.0031 (14)	−0.0003 (13)	−0.0080 (14)
C28	0.0264 (15)	0.0236 (14)	0.0320 (15)	−0.0001 (11)	0.0036 (12)	−0.0045 (11)
O1	0.0362 (11)	0.0281 (11)	0.0372 (11)	0.0063 (9)	−0.0059 (9)	−0.0082 (9)
O2	0.0262 (11)	0.0669 (16)	0.0277 (11)	−0.0045 (10)	0.0008 (9)	−0.0187 (10)
O3	0.0271 (12)	0.0528 (15)	0.0660 (16)	−0.0030 (11)	−0.0029 (11)	−0.0014 (12)
O4	0.0358 (13)	0.0807 (19)	0.0625 (17)	−0.0016 (13)	−0.0140 (12)	−0.0126 (14)
O5	0.0427 (13)	0.0291 (12)	0.0562 (14)	0.0126 (9)	−0.0158 (11)	−0.0028 (10)
O6	0.0261 (12)	0.0661 (15)	0.0452 (13)	0.0183 (10)	0.0115 (10)	0.0271 (11)
O7	0.0251 (10)	0.0284 (10)	0.0404 (11)	0.0060 (8)	0.0058 (8)	0.0038 (9)
O8	0.0313 (11)	0.0269 (10)	0.0490 (13)	0.0038 (8)	0.0139 (9)	0.0085 (9)
O9	0.0336 (11)	0.0345 (11)	0.0276 (10)	0.0028 (9)	−0.0064 (8)	0.0035 (9)
O10	0.0444 (13)	0.0315 (11)	0.0424 (12)	−0.0071 (9)	0.0161 (10)	−0.0033 (9)
O11	0.0248 (10)	0.0259 (10)	0.0462 (12)	0.0007 (8)	0.0009 (9)	0.0057 (9)
O12	0.0240 (11)	0.0488 (13)	0.0428 (12)	−0.0061 (9)	−0.0045 (9)	−0.0011 (10)
O13	0.354 (10)	0.169 (6)	0.185 (6)	0.015 (6)	−0.141 (6)	0.009 (5)

### Geometric parameters (Å, º)

**Table:**

Pr1—O9^i^	2.3983 (18)	C12—C26	1.389 (4)
Pr1—O5	2.4105 (19)	C12—H12	0.9300
Pr1—O12^ii^	2.412 (2)	C13—C21	1.378 (4)
Pr1—O8^iii^	2.4692 (19)	C13—O6	1.379 (3)
Pr1—O11	2.4719 (18)	C14—C25	1.380 (4)
Pr1—O10	2.5152 (19)	C14—H14	0.9300
Pr1—O1	2.5163 (19)	C15—O4	1.216 (4)
Pr1—O7^iii^	2.6534 (19)	C15—O3	1.320 (4)
C1—O5	1.252 (3)	C16—C24	1.375 (4)
C1—O12	1.252 (3)	C16—C17	1.390 (4)
C1—C2	1.496 (4)	C16—H16	0.9300
C2—C18	1.375 (4)	C17—O2	1.379 (4)
C2—C9	1.381 (4)	C17—C27	1.380 (4)
C3—C14	1.382 (4)	C18—C20	1.384 (5)
C3—C10	1.385 (4)	C18—H18	0.9300
C3—C4	1.497 (4)	C19—H19	0.9300
C4—O9	1.254 (3)	C20—H20	0.9300
C4—O1	1.266 (3)	C21—H21	0.9300
C5—C20	1.353 (5)	C22—C26	1.386 (4)
C5—C19	1.371 (5)	C22—H22	0.9300
C5—O6	1.404 (3)	C23—C27	1.380 (5)
C6—C24	1.385 (5)	C23—H23	0.9300
C6—C23	1.386 (4)	C24—H24	0.9300
C6—C15	1.480 (4)	C25—H25	0.9300
C7—C22	1.381 (4)	C26—C28	1.481 (4)
C7—C13	1.381 (4)	C27—H27	0.9300
C7—H7	0.9300	C28—O7	1.262 (3)
C8—C11	1.367 (5)	C28—O8	1.273 (3)
C8—C25	1.369 (4)	O3—H3	0.8200
C8—O2	1.406 (3)	O10—H10A	0.8499
C9—C19	1.381 (4)	O10—H10B	0.8500
C9—H9	0.9300	O11—H11B	0.8444
C10—C11	1.385 (4)	O11—H11A	0.8444
C10—H10	0.9300	O13—H13B	0.8542
C11—H11	0.9300	O13—H13A	0.8500
C12—C21	1.381 (4)		
			
O9^i^—Pr1—O5	153.69 (8)	O6—C13—C7	116.0 (3)
O9^i^—Pr1—O12^ii^	88.26 (7)	C25—C14—C3	120.8 (3)
O5—Pr1—O12^ii^	107.34 (7)	C25—C14—H14	119.6
O9^i^—Pr1—O8^iii^	74.83 (7)	C3—C14—H14	119.6
O5—Pr1—O8^iii^	128.11 (7)	O4—C15—O3	123.6 (3)
O12^ii^—Pr1—O8^iii^	78.82 (7)	O4—C15—C6	122.1 (3)
O9^i^—Pr1—O11	70.57 (6)	O3—C15—C6	114.2 (3)
O5—Pr1—O11	83.78 (7)	C24—C16—C17	118.9 (3)
O12^ii^—Pr1—O11	136.39 (7)	C24—C16—H16	120.5
O8^iii^—Pr1—O11	127.57 (6)	C17—C16—H16	120.5
O9^i^—Pr1—O10	85.53 (7)	O2—C17—C27	123.8 (3)
O5—Pr1—O10	79.66 (7)	O2—C17—C16	115.5 (3)
O12^ii^—Pr1—O10	71.94 (7)	C27—C17—C16	120.7 (3)
O8^iii^—Pr1—O10	145.13 (7)	C2—C18—C20	120.2 (3)
O11—Pr1—O10	68.85 (6)	C2—C18—H18	119.9
O9^i^—Pr1—O1	107.40 (6)	C20—C18—H18	119.9
O5—Pr1—O1	69.94 (7)	C5—C19—C9	118.9 (3)
O12^ii^—Pr1—O1	148.79 (7)	C5—C19—H19	120.6
O8^iii^—Pr1—O1	79.55 (7)	C9—C19—H19	120.6
O11—Pr1—O1	74.82 (7)	C5—C20—C18	120.0 (3)
O10—Pr1—O1	134.54 (7)	C5—C20—H20	120.0
O9^i^—Pr1—O7^iii^	123.84 (6)	C18—C20—H20	120.0
O5—Pr1—O7^iii^	81.88 (7)	C13—C21—C12	119.2 (3)
O12^ii^—Pr1—O7^iii^	71.13 (7)	C13—C21—H21	120.4
O8^iii^—Pr1—O7^iii^	50.58 (6)	C12—C21—H21	120.4
O11—Pr1—O7^iii^	152.06 (6)	C7—C22—C26	120.5 (3)
O10—Pr1—O7^iii^	131.07 (6)	C7—C22—H22	119.7
O1—Pr1—O7^iii^	77.77 (6)	C26—C22—H22	119.7
O5—C1—O12	122.6 (3)	C27—C23—C6	120.6 (3)
O5—C1—C2	117.8 (2)	C27—C23—H23	119.7
O12—C1—C2	119.7 (2)	C6—C23—H23	119.7
C18—C2—C9	118.8 (3)	C16—C24—C6	121.2 (3)
C18—C2—C1	121.0 (3)	C16—C24—H24	119.4
C9—C2—C1	120.2 (2)	C6—C24—H24	119.4
C14—C3—C10	118.9 (3)	C8—C25—C14	118.9 (3)
C14—C3—C4	120.3 (2)	C8—C25—H25	120.5
C10—C3—C4	120.8 (2)	C14—C25—H25	120.5
O9—C4—O1	122.7 (2)	C22—C26—C12	118.8 (3)
O9—C4—C3	118.9 (2)	C22—C26—C28	121.5 (3)
O1—C4—C3	118.4 (2)	C12—C26—C28	119.7 (3)
C20—C5—C19	121.1 (3)	C23—C27—C17	119.5 (3)
C20—C5—O6	119.2 (3)	C23—C27—H27	120.3
C19—C5—O6	119.6 (3)	C17—C27—H27	120.3
C24—C6—C23	119.0 (3)	O7—C28—O8	119.9 (3)
C24—C6—C15	119.3 (3)	O7—C28—C26	121.8 (2)
C23—C6—C15	121.7 (3)	O8—C28—C26	118.2 (2)
C22—C7—C13	119.8 (3)	O7—C28—Pr1^iv^	64.72 (15)
C22—C7—H7	120.1	O8—C28—Pr1^iv^	56.40 (14)
C13—C7—H7	120.1	C26—C28—Pr1^iv^	166.2 (2)
C11—C8—C25	121.7 (3)	C4—O1—Pr1	120.84 (17)
C11—C8—O2	120.2 (3)	C17—O2—C8	118.1 (2)
C25—C8—O2	118.1 (3)	C15—O3—H3	109.5
C19—C9—C2	121.0 (3)	C1—O5—Pr1	123.47 (18)
C19—C9—H9	119.5	C13—O6—C5	117.6 (2)
C2—C9—H9	119.5	C28—O7—Pr1^iv^	89.80 (16)
C3—C10—C11	120.5 (3)	C28—O8—Pr1^iv^	98.18 (16)
C3—C10—H10	119.8	C4—O9—Pr1^i^	170.37 (18)
C11—C10—H10	119.8	Pr1—O10—H10A	109.2
C8—C11—C10	119.0 (3)	Pr1—O10—H10B	109.4
C8—C11—H11	120.5	H10A—O10—H10B	109.5
C10—C11—H11	120.5	Pr1—O11—H11B	116.8
C21—C12—C26	121.1 (3)	Pr1—O11—H11A	122.6
C21—C12—H12	119.5	H11B—O11—H11A	106.3
C26—C12—H12	119.5	C1—O12—Pr1^ii^	172.3 (2)
C21—C13—O6	123.3 (3)	H13B—O13—H13A	99.7
C21—C13—C7	120.7 (3)		
			
O5—C1—C2—C18	−163.0 (3)	C7—C22—C26—C12	−0.5 (5)
O12—C1—C2—C18	16.2 (4)	C7—C22—C26—C28	179.8 (3)
O5—C1—C2—C9	17.7 (4)	C21—C12—C26—C22	−0.4 (5)
O12—C1—C2—C9	−163.1 (3)	C21—C12—C26—C28	179.2 (3)
C14—C3—C4—O9	−6.8 (4)	C6—C23—C27—C17	0.7 (5)
C10—C3—C4—O9	175.4 (3)	O2—C17—C27—C23	178.2 (3)
C14—C3—C4—O1	172.3 (3)	C16—C17—C27—C23	−0.1 (5)
C10—C3—C4—O1	−5.6 (4)	C22—C26—C28—O7	17.2 (4)
C18—C2—C9—C19	0.6 (5)	C12—C26—C28—O7	−162.4 (3)
C1—C2—C9—C19	179.9 (3)	C22—C26—C28—O8	−164.6 (3)
C14—C3—C10—C11	−2.3 (5)	C12—C26—C28—O8	15.7 (4)
C4—C3—C10—C11	175.5 (3)	C22—C26—C28—Pr1^iv^	131.9 (7)
C25—C8—C11—C10	1.5 (5)	C12—C26—C28—Pr1^iv^	−47.8 (9)
O2—C8—C11—C10	180.0 (3)	O9—C4—O1—Pr1	−16.0 (4)
C3—C10—C11—C8	0.2 (5)	C3—C4—O1—Pr1	165.01 (17)
C22—C7—C13—C21	−1.1 (5)	O9^i^—Pr1—O1—C4	21.5 (2)
C22—C7—C13—O6	179.5 (3)	O5—Pr1—O1—C4	173.8 (2)
C10—C3—C14—C25	2.9 (5)	O12^ii^—Pr1—O1—C4	−95.6 (2)
C4—C3—C14—C25	−175.0 (3)	O8^iii^—Pr1—O1—C4	−48.8 (2)
C24—C6—C15—O4	2.3 (5)	O11—Pr1—O1—C4	85.0 (2)
C23—C6—C15—O4	−176.4 (3)	O10—Pr1—O1—C4	122.68 (19)
C24—C6—C15—O3	−176.6 (3)	O7^iii^—Pr1—O1—C4	−100.5 (2)
C23—C6—C15—O3	4.7 (5)	C28^iii^—Pr1—O1—C4	−74.6 (2)
C24—C16—C17—O2	−178.6 (3)	C27—C17—O2—C8	8.6 (4)
C24—C16—C17—C27	−0.2 (5)	C16—C17—O2—C8	−173.0 (3)
C9—C2—C18—C20	−1.7 (5)	C11—C8—O2—C17	73.5 (4)
C1—C2—C18—C20	179.0 (3)	C25—C8—O2—C17	−108.0 (3)
C20—C5—C19—C9	−1.6 (5)	O12—C1—O5—Pr1	−4.2 (4)
O6—C5—C19—C9	−177.9 (3)	C2—C1—O5—Pr1	174.99 (17)
C2—C9—C19—C5	1.0 (5)	O9^i^—Pr1—O5—C1	−115.7 (2)
C19—C5—C20—C18	0.6 (5)	O12^ii^—Pr1—O5—C1	8.3 (3)
O6—C5—C20—C18	176.9 (3)	O8^iii^—Pr1—O5—C1	97.6 (2)
C2—C18—C20—C5	1.1 (6)	O11—Pr1—O5—C1	−128.5 (2)
O6—C13—C21—C12	179.5 (3)	O10—Pr1—O5—C1	−58.9 (2)
C7—C13—C21—C12	0.2 (5)	O1—Pr1—O5—C1	155.5 (3)
C26—C12—C21—C13	0.6 (5)	O7^iii^—Pr1—O5—C1	75.6 (2)
C13—C7—C22—C26	1.3 (5)	C28^iii^—Pr1—O5—C1	82.5 (2)
C24—C6—C23—C27	−0.9 (5)	C21—C13—O6—C5	5.7 (5)
C15—C6—C23—C27	177.9 (3)	C7—C13—O6—C5	−175.0 (3)
C17—C16—C24—C6	−0.1 (5)	C20—C5—O6—C13	92.8 (4)
C23—C6—C24—C16	0.6 (5)	C19—C5—O6—C13	−90.9 (4)
C15—C6—C24—C16	−178.2 (3)	O8—C28—O7—Pr1^iv^	−12.0 (3)
C11—C8—C25—C14	−1.0 (5)	C26—C28—O7—Pr1^iv^	166.1 (2)
O2—C8—C25—C14	−179.5 (3)	O7—C28—O8—Pr1^iv^	13.1 (3)
C3—C14—C25—C8	−1.2 (5)	C26—C28—O8—Pr1^iv^	−165.1 (2)

Symmetry codes: (i) −*x*, −*y*+1, −*z*; (ii) −*x*, −*y*, −*z*; (iii) *x*−1/2, *y*+1/2, *z*; (iv) *x*+1/2, *y*−1/2, *z*.

### Hydrogen-bond geometry (Å, º)

**Table:**

*D*—H···*A*	*D*—H	H···*A*	*D*···*A*	*D*—H···*A*
O3—H3···O1^v^	0.82	2.02	2.822 (3)	166
O10—H10*B*···O2^vi^	0.85	2.09	2.880 (3)	154
O11—H11*A*···O4^vii^	0.84	1.84	2.683 (3)	176
O11—H11*B*···O8^viii^	0.84	1.89	2.707 (3)	163
C9—H9···O3^vii^	0.93	2.48	3.337 (4)	153
C25—H25···O13^iii^	0.93	2.59	3.454 (7)	155

Symmetry codes: (iii) *x*−1/2, *y*+1/2, *z*; (v) −*x*+1/2, *y*+1/2, −*z*+1/2; (vi) *x*, −*y*+1, *z*−1/2; (vii) −*x*+1/2, *y*−1/2, −*z*+1/2; (viii) −*x*+1/2, −*y*+1/2, −*z*.

## References

[bb1] Bruker (1997). *SAINT*, *SMART* and *SADABS* Bruker AXS Inc., Madison, Wisconsin, USA.

[bb2] Li, X. X., Wei, Z. Q., Yue, S. T., Wang, N., Mo, H. H. & Liu, Y. L. (2011). *J. Chem. Crystallogr.* **41**, 757–761.

[bb3] Lin, Y. W., Jian, B. R., Huang, S. C., Huang, C. H. & Hsu, K. F. (2010). *Inorg. Chem.*, **49**, 2316–2324. 10.1021/ic9021992 20121147

[bb4] Łyszczek, R. & Mazur, L. (2012). *Inorg. Chem. Commun.* **15**, 121–125.

[bb5] Sheldrick, G. M. (2008). *Acta Cryst.* A**64**, 112–122. 10.1107/S0108767307043930 18156677

[bb6] Sun, C. Y., Zheng, X. B., Li, L. C. & Jin, L. P. (2009). *Inorg. Chim. Acta*, **362**, 325–330.

[bb7] Thirumurugan, A. & Natarajan, S. (2004). *Eur. J. Inorg. Chem.* pp. 762–770.

[bb8] Wang, Y. B., Sun, C. Y., Zheng, X. J., Gao, S., Lu, S. Z. & Jin, L. P. (2005). *Polyhedron*, **24**, 823–830.

[bb9] Wang, Y. B., Wang, Z. M., Yan, C. H. & Jin, L. P. (2004). *J. Mol. Struct.* **692**, 177–186.

[bb10] Xu, J., Su, W. P. & Hong, M. C. (2011). *Inorg. Chem. Commun.* **14**, 1794–1797.

[bb11] Zhang, J. J., Hu, S. M., Xiang, S. C., Wang, L. S., Li, Y. M., Zhang, H. S. & Wu, X. T. (2005). *J. Mol. Struct.* **748**, 129–136.

